# Prevalence of *Cryptococcus gattii* in Ugandan HIV-infected patients presenting with cryptococcal meningitis

**DOI:** 10.1371/journal.pone.0270597

**Published:** 2022-07-15

**Authors:** Abel Wembabazi, Dianah Rhoda Nassozi, Enid Akot, Timothy Isaac Ochola, Prosper Tom Kweka, Nelson Tom Katamu, David Meya, Beatrice Achan

**Affiliations:** 1 School of Health Sciences, College of Health Sciences, Makerere University, Kampala, Uganda; 2 School of Medicine, College of Health Sciences, Makerere University, Kampala, Uganda; 3 School of Biomedical Sciences, College of Health Sciences, Makerere University, Kampala, Uganda; 4 Infectious Diseases Institute, College of Health Sciences, Makerere University, Kampala, Uganda; 5 Department of Medicine, College of Health Sciences, Makerere University, Kampala, Uganda; 6 Department of Medicine, University of Minnesota, Minneapolis, Minnesota, United States of America; 7 Department of Medical Microbiology, College of Health Sciences, Makerere University, Kampala, Uganda; Yonsei University, REPUBLIC OF KOREA

## Abstract

**Introduction:**

Cryptococcal meningitis (CM) is a life threatening disease and leading cause of opportunistic fungal-related mortality in HIV/AIDS. Most CM infections are caused by *C*. *neoformans* species complexes but the prevalence of *Cryptococcus gattii* species complexes in Uganda is unknown however, it is known in a few other parts of Africa. We estimated the prevalence of *C*. *gattii* in patients living with HIV and a diagnosis of cryptococcal meningitis in Uganda.

**Methods:**

*Cryptococcus* isolates (n = 200) obtained from cerebrospinal fluid of patients with CM recruited at the Infectious Diseases Institute, Kampala, Uganda, were tested by phenotypic methods. The *Cryptococcus* isolates were sub-cultured on Sabouraud Dextrose Agar plates for 48 hours. The yeast colonies were examined by India ink stain, urea hydrolysis, and *C*. *gattii* was identified by blue pigmentation on CGB agar. The results were analyzed for frequency of *C*. *gattii*. Patient demographic characteristics were collected from the case record forms.

**Results:**

From the 200 patients’ case record forms, 87 (43.5%) were female and 113 (56.5%) were male. The median age was 35 (19–64) years. Most patients, 93% (187/200) were from Central Uganda in the districts of Kampala and Wakiso. 97.51% (157/161) of the patients had absolute CD4 lymphocyte counts of less than 200 cells per cubic millimeter; 1.86% (3/161) 200–350 cells per cubic millimeter and 0.62% (1/161) above 500 cells per cubic millimeter. 45.4% (74/163) were not yet on HAART and 54.6% (89/163) were on HAART. 66.7% (58/87) had poor adherence to HAART treatment and 33.3% (29/87) had reported good adherence to HAART treatment. A total of 200 clinical isolates of *Cryptococcus* isolates were tested. No (0% (0/200) *C*. *gattii* was identified among the *Cryptococcus* isolates.

**Conclusion:**

In this study among patients living with HIV and a diagnosis of cryptococcal meningitis in Uganda, we found no *C*. *gattii* infections.

## Introduction

Globally, almost 1 million people succumb to HIV/AIDS associated infections every year [1]. Of the 38 million people living with HIV/AIDS globally, 69% live in sub-Saharan Africa [2].

Cryptococcal meningitis (CM) is a life threatening disease and leading cause of opportunistic fungal-related mortality in HIV/AIDS. Approximately 223,100 infections are documented annually, 70% of which occur in sub-Saharan Africa [3]. It is estimated that CM accounts for 60% of life-threatening meningitis among people living with HIV/AIDS in Uganda [4]. With no treatment, the two-week mortality rate associated with acute CM is almost always 100% [3]. CM is caused by two closely related species complexes of *Cryptococcus*. The *C*. *neoformans* and *C*. *gattii* species complexes that belong to the phylum Basidiomycota [5]. The taxonomy of *C*. *gattii/C*. *neoformans* species complexes has had a lot of debate from fungal taxonomists. However, advances in phylogenetic and genotypic studies now identify them as separate species complexes; *C*. *neoformans* previously known as *C*. *neoformans* var. *neoformans* and *C*. *gattii* formerly known as *C*. *neofarmans var*. *gattii* [6]. These species complexes differ from other pathogenic yeasts such as *Candida* species by the presence of a polysaccharide capsule, formation of melanin, and urease activity, which all function as virulence determinant factors [5].

*C*. *neoformans* is globally distributed and causes CM in immunocompromised individuals for example, in patients with cancer, solid organ transplant, and HIV/AIDS [7] The sibling species *Cryptococcus gattii* has traditionally shown a geographic restriction to tropical and subtropical regions [8, 9], ecological localization to the red gum trees, *Eucalyptus* species [10–12] and pathogenicity in immunocompetent individuals [13–16].

However, recent literature showed an outbreak of *C*. *gattii* in the temperate Vancouver island [17] and, in tropical Africa, it was isolated from other tree types in Zambia [18]. The prevalence of *C*. *gattii* is unknown in Uganda. To our knowledge, there is no related study about *C*. *gattii* infections done up to date in Uganda. In this study, we sought to determine the prevalence of *C*. *gattii* in patients with cryptococcal meningitis at the Infectious Diseases Institute (IDI), Kampala, Uganda.

## Materials and methods

### Ethics approval and consent to participate

The study was approved by the School of Biomedical Sciences’ Higher Degrees’ Research and Ethics Committee (SBS-HDREC), College of Health Sciences, Makerere University (Reference number SBS-REC 839). Waiver of consent was granted to use the *Cryptococcus* isolates already available. The Uganda National Council for Science and Technology, UNCST registration numbers of the parent studies, HS213ES, HS2317, and HS2351 are included. Additional administrative permissions were sought from the Clinical Microbiology and Mycology laboratories, College of Health Sciences, Makerere University.

### Study design and duration

The retrospective study was conducted from March 2021 to August 2021 inclusive.

### Study site

The study was conducted at the Clinical Microbiology, and Mycology laboratories at the College of Health Sciences (MakCHS), Makerere University, Kampala, Uganda.

### Target population

*Cryptococcus* clinical isolates from the cerebrospinal fluid of patients with cryptococcal meningitis recruited at IDI, Kampala, Uganda.

### Inclusion criteria

*Cryptococcus clinical* isolates from the cerebrospinal fluid of patients with cryptococcal meningitis recruited at IDI, Kampala Uganda, stored between 2018 to 2021.

### Sample size

A total of two hundred (n = 200) clinical *Cryptococcus* isolates were obtained from the cerebrospinal fluid of CM patients from the Infectious Diseases Institute, Kampala, Uganda. A positive control for *C*. *neoformans* was included however; we did not have a positive control for *C*. *gattii*. The sample size was determined using Kish and Leslie formula (Kish *et al*., 1965), using an estimated environmental prevalence of *C*. *gattii* of 15% from Zambia [18] and standard deviation at 95% Confidence Interval (1.960).

### Sampling technique

Consecutive sampling was performed until the sample size of 200 *Cryptococcus* isolates was achieved. The isolates were selected from those stored as follows; 16 isolates from 2018, 65 isolates from 2019, 89 isolates from 2020 and 30 from 2021.

### Data collection procedures

#### Laboratory methods

Stored isolates of *Cryptococcus* were used for this study. The *Cryptococcus* isolates in the glycerol stocks were retrieved from the—80°C freezers, sub-cultured on Sabouraud Dextrose Agar plate. After 48 hours, the yeast colonies were identified phenotypically. Briefly, the colonies were stained with India ink and examined by x40 objective. The colonies were further inoculated onto urea agar for 48 hours. Lastly, pure, single colonies isolates were streaked on CGB agar plate using a loop. CGB agar plate preparation was done as described here [19].

#### Case record forms

Case record forms (CRF) were used for data associated with the host factors of the persons from where the isolates were obtained. The CRF include demographic data, HIV status, absolute CD4 lymphocyte counts, initiation and adherence to Highly active antiretroviral therapy (HAART) and CM diagnosis.

## Results

From the 200 corresponding case record forms, 87 (43.5%) were female and 113 (56.5%) were male. The median age was 35 (interquartile range, 19–64) years. Most participants were from central Uganda, 93.5% (187) in the districts of Kampala and Wakiso. Most patients had absolute CD4 lymphocyte counts of less than 200 cells per cubic millimeter (97.51%; 157/161), 1.86% (3/161) had absolute CD4 lymphocyte counts in range of 200–350 cells per cubic millimeter and 0.62% (1/161) had absolute CD4 lymphocyte counts of above 500 cells per cubic millimeter. HIV viral loads for six patients were captured, and the median viral load was 56,522 cells per milliliter (range 5,015–198,700 cells per milliliter). 45.4% (74/163) were not yet on HAART and 54.6% (89/163) were on HAART. The HAART combinations included NRTI such as 3TC, ABC, TDF and AZT among others. 66.7% (58/87) had poor adherence to HAART treatment and 33.3% (29/87) had reported good adherence to HAART treatment (Table 1).

**Table 1:** Patients’ demographic characteristics and HIV related data.

Patients’ demographics		Patients’ HIV related data			
Gender		Initiation on HAART			
Male	113/200 (56.5%)	Naïve			74/163 (45.4%)
Female	87/200 (43.5)	Yes			89/163 (54.6%)
**Age**			Adherence to HAART	Good	29/87 (33.3%)
10–19	2/200 (1%)			Poor	58/87 (66.7%)
20–29	47/200 (23.5%)			Unknown	2*
30–39	90/200 (45%)	**Absolute CD4 counts (cells/mm^3)**			
40–49	45/200 (22.5%)	<200		157/161 (97.52%)	
50–59	12/200 (6%)	200–349		3/161 (1.86%)	
60–69	4/200 (2%)	350–499		0/161 (0%)	
**Home Region**		>500		1/161 (0.62%)	
Central	187/200 (93.5%)
Western	4/200 (2%)
Eastern	4/200 (2%)
Northern	0/200 (0%)
Not shown	5/200 (2.5%)

* 2 patients on HAART had unknown adherence

A total of two hundred (n = 200) *Cryptococcus* isolates were tested to identify *C*. *gattii*. No positive controls for *C*. *gattii* strains were included in the study. All the isolates grew on Sabouraud Dextrose agar plates producing white colonies. All the colonies were positive when examined by India ink stain by exhibiting a halo around the cell against the black background using x40 objective. Additionally, all isolates were urease positive by turning the agar from yellow to pink after incubation. However, all isolates had a negative reaction on the CGB agar as there was no color change in the medium. Therefore, there was no *Cryptococcus gattii* strains among the Cryptococcal isolates tested.

## Discussion

We found no *Cryptococcus gattii* among the clinical isolates sampled from the cerebrospinal fluid of patients with cryptococcal meningitis at the IDI, suggesting that the great majority, if not all infections causing cryptococcal meningitis result from *Cryptococcus neoformans* and not *Cryptococcus gattii* in this population.

However, some studies have reported clinical cases of *C*. *gattii* infections in the recent past both from seronegative and seropositive HIV patients. In South Africa, 5/276 (1.8%) prevalence was reported inclusive of one HIV seronegative patient [20]. Other studies included one case from a leukemic patient in Congo [21], 4/70 (5.7%) from HIV seropositive patients in Kenya [22], 8/499 (1.6%) from HIV seropositive in Rwanda [23], 11/66 (16.7%) from HIV seropositive patients in Zimbabwe [24].

Our negative findings could be attributed to: the low diagnostic accuracy of phenotypic based tests used such as the CGB agar as suggested by some studies that reported some false positives in identification of *C*. *gattii* [25]. However, due to limited resources within the scope of this project, CGB agar remained the option because of some of its strengths like being reliable, affordable and easy to work with as indicated by some studies [19, 26]. Secondly, late presentation of our patients in advanced clinical HIV stage 4 with CM at CD4 cell count <200 indicates severe immunosuppression [27], yet *C*. *gattii* infections are more prevalent in immune competent persons [13, 14, 16]. Therefore, we believe that our study population which was comprised mostly of immune compromised individuals was not appropriate for isolating *C*. *gattii* and likely CM infections in our patients are due to *C*. *neoformans*. Lastly, lack of clinical and environmental information on *C*. *gattii* incidences in Uganda could have affected the precision of our results. To our knowledge, there is no related study about *C*. *gattii* done up to date.

Our study limitation included a small sample size, which may not represent the entire geographical locations where *C*. *gattii* could be found in Uganda. We recommend environmental sampling for *C*. *gattii* to further determine its presence in Uganda.

## Supporting information

S1 Data (XLSX)
